# TLR-3 Stimulation Skews M2 Macrophages to M1 Through IFN-αβ Signaling and Restricts Tumor Progression

**DOI:** 10.3389/fimmu.2018.01650

**Published:** 2018-07-19

**Authors:** Aurobind Vidyarthi, Nargis Khan, Tapan Agnihotri, Shikha Negi, Deepjyoti K. Das, Mohammad Aqdas, Deepyan Chatterjee, Oscar R. Colegio, Manoj K. Tewari, Javed N. Agrewala

**Affiliations:** ^1^Immunology Laboratory, CSIR-Institute of Microbial Technology, Chandigarh, India; ^2^Department of Dermatology, Yale University School of Medicine, New Haven, CT, United States; ^3^Department of Neurosurgery, Postgraduate Institute of Medical Education and Research, Chandigarh, India; ^4^Indian Institute of Technology, Ropar, India

**Keywords:** tumor-associated macrophages, IFN-αβ, TLR-3, tumor microenvironment, Tim-3, CD206

## Abstract

During tumor progression, macrophages shift their protective M1-phenotype to pro-tumorigenic M2-subtype. Therefore, conversion of M2 to M1 phenotype may be a potential therapeutic intervention. TLRs are important pathogen recognition receptors expressed by cells of the immune system. Recently, a crucial role of TLR-3 has been suggested in cancer. Consequently, in the current study, we defined the role of TLR-3 in the reversion of M2-macrophages to M1. We analyzed the role of TLR-3 stimulation for skewing M2-macrophages to M1 at mRNA and protein level through qRT-PCR, flow cytometry, western blotting, and ELISA. The effectiveness of TLR-3L stimulation to revert M2-macrophages to M1 was evaluated in the murine tumor model. To determine the role of IFN-αβ signaling *in vitro* and *in vivo*, we used *Ifnar1*^−/−^ macrophages and anti-IFN-αβ antibodies, respectively. We observed upregulation of M1-specific markers MHC-II and costimulatory molecules like CD86, CD80, and CD40 on M2-macrophages upon TLR-3 stimulation. In contrast, reduced expression of M2-indicators CD206, *Tim-3*, and pro-inflammatory cytokines was noticed. The administration of TLR-3L in the murine tumor reverted the M2-macrophages to M1-phenotype and regressed the tumor growth. The mechanism deciphered for macrophage reversion and controlling the tumor growth is dependent on IFN-αβ signaling pathway. The results indicate that the signaling through TLR-3 is important in protection against tumors by skewing M2-macrophages to protective M1-subtype.

## Introduction

Macrophages play a complex role in tumor biology. Their presence within the tumor microenvironment can be correlated with an increase in the size of the tumor (1). Accumulating evidence suggest that a dynamic change in the phenotype of macrophages is involved in tumor initiation, progression, and metastasis (2, 3). During early phase of tumor progression, macrophages acquire M1 subtype, which are characterized as a pro-inflammatory phenotype, display microbicidal and antitumor activity (4). In later stage of neoplastic transformation, tumor milieu promotes the polarization of macrophages toward M2 subtype, which support tissue repair, matrix remodeling, angiogenesis, and suppression of antitumor immunity. This suggests that M2 macrophages support tumorigenesis (5). Noteworthy, depending on the tumor microenvironment, macrophages acquire various phenotypes. Experimental and clinical evidence suggest that M2 macrophages provide tropic support to tumors. However, with the change in the milieu, tumor progression can be controlled. Further, targeting M2 macrophages or their unique signaling pathways could be a therapeutic intervention to control the tumor growth (6). Moreover, conversion of macrophages to an M2 phenotype *in vivo* can result in evasion of tumor immune-surveillance. Many factors are known to contribute to the diversity of macrophages function. The synergistic effects of different cytokines drive the specialized and polarized functional properties of macrophages. Classically activated M1 macrophages are induced by Lipopolysaccharide (LPS) and IFN-γ. Signaling delivered through IL-4 and IL-13 induces an alternative M2 form of macrophage activation. M2 macrophages do not constitute a uniform population and often are subdivided into M2a, M2b, and M2c categories. M2a form is driven by IL-4 and IL-13, whereas M2c require TGF-β and IL-10 for acquiring their classical features. In addition to cytokines, signaling delivered through ligands of innate immunity also play a crucial role in changing the profile of macrophages. In the past, Toll like receptors (TLRs) agonists have been used as an adjuvant for the treatment of cancer (7). TLR-3 is copiously expressed on different subsets of macrophages. Unfortunately, not much has been known about the role of TLR-3 in skewing tumor-associated M2 macrophages to tumor-protective M1 subtype.

Therefore, the current study was designed to explore the importance of signaling through TLR-3 in reverting pro-tumorigenic M2 subtype to antitumorigenic M1 macrophages and subsequently studying its impact in controlling the tumor growth. Interestingly, we observed that signaling delivered through TLR-3 efficiently polarized macrophages from M2 to M1 phenotype. Further, it substantially restricted the growth of tumors. Thus, this study signifies an important therapeutic role of TLR-3 in treating tumors.

## Materials and Methods

### Animals

C57BL/6 mice, 6–8 weeks (20 ± 2 g) were procured from the CSIR-Institute of Microbial Technology, Chandigarh, India. C57BL/6 wild-type and *ifnar^−^*^/^*^−^* mice were a kind gift from Dr. Oscar R Colegio, Department of Dermatology, Yale University School of Medicine, New Haven, CT, USA and Dr. Priti Kumar, Department of Internal Medicine, Section of Infectious Diseases, Yale University School of Medicine, New Haven, CT, USA, respectively.

### Abs and Reagents

All the recombinant cytokines, Abs, and ELISA reagents were purchased from BD Biosciences (San Diego, CA, USA) or unless mentioned. TLR-3L [poly (I:C); Catalog: tlrl-picw] was procured from Invivogen (San Diego, CA, USA).

### Cell Lines and Culture of Bone Marrow-Derived Macrophages (BMDM)

MC38 was a kind gift from Dr. G. Shurin, University of Pittsburgh and THP-1 was a kind gift from Dr. Oscar R Colegio, Yale University. MC38 and THP-1 cell lines were maintained in DMEM and RPMI-1640 (Invitrogen Life Technologies, Eugene, OR, USA), respectively. Both the media were supplemented with 10% FBS (GIBCO, Grand Island, NY, USA), with penicillin (100 U/ml), streptomycin (100 mg/ml), and l-glutamine (100 mM). BMDM were cultured by flushing bone marrow cells (BMs) aseptically from femurs and tibia of mice. Cells were grown in complete RPMI media as mentioned above and supplemented with L929 (20%) Supernatant (SN), as a source of macrophage-colony stimulating factor (M-CSF) (2). Cultures were maintained in a humidified atmosphere at 5% CO_2_/37°C. The medium was replenished on day 3. On day 6, macrophages were harvested.

### Polarization of Macrophages

Macrophages were stimulated with LPS (100 ng/ml) and IFN-γ (20 ng/ml) for their polarization toward M1 subtypes; IL-4 (10 ng/ml) and IL-13 (10 ng/ml) for their polarization toward M2a subtypes; TGF-β (10 ng/ml) and IL-10 (10 ng/ml) for their polarization toward M2c subtypes for 24 h in complete RPMI-media. Later, cells were washed and stimulated with TLR-3 ligand (TLR-3L) poly (I:C) in complete RPMI media for 24 h. For neutralization experiments, neutralizing Abs against IL-6 (20 µg/ml), IL-12 (20 µg/ml), TNF-α (10 µg/ml), IFN-γ (10 µg/ml), and blocking Ab for IFN-αβR (20 µg/ml) were added in the cultures along with TLR-3L for 24 h.

### Immunofluorescent Staining

Polarized macrophages (M1, M2a, M2c subtypes) stimulated with poly (I:C), as mentioned above, were resuspended in FACS buffer (FCS-2%, 2 mM sodium azide in PBS). To inhibit non-specific staining, cells were incubated with anti-CD16/32 Ab for 25 min at 4°C. Later, cells were stained with fluorochrome conjugated Abs specific for mouse F4/80, CD80, CD40, PDL-1, CD86, MHC-II, TIM-3, CD206, or isotype-matched control Abs, at a recommended concentration (0.5 μg/10^6^ cells). The cells were fixed with 1× paraformaldehyde. Regular steps of washing were followed at each step (8). Data were collected using BD FACS Aria flow cytometer and analyzed with BD DIVA software and FlowJo software.

### Propidium Iodide (PI) and Annexin V Assays

Polarized macrophages were stimulated for 24 h with poly (I:C) as mentioned above. After 24 h, stimulated macrophages were harvested and resuspended in the 100 µl of binding buffer [0.01 M HEPES (pH7.4), 0.14 M NaCl and 2.5 mM CaCl2] containing FITC-conjugated annexin V (5 μl/tube) and 5 µl of PI (50 µg/ml) and incubated in the dark for 15 min at 37°C. Later, binding buffer (400 µl) was added and cells were acquired immediately using BD FACS Aria flow cytometer, and data were analyzed using BD DIVA software (9).

### Cytokines Estimation

Polarized macrophages were stimulated with poly (I:C) for 24 h as mentioned above. After 24 h, supernatants were collected for detection of cytokines, *viz*, IL-6, IL-12, TNF-α, IL-1β, and IL-10 by standard ELISA according to manufacturer’s instruction (BD Biosciences San Diego, CA, USA). For IFN-β ELISA VeriKine ELISA Kit and for IFN-α Thermo Fisher Scientific ELISA kit were used.

### Antigen Uptake

Polarized macrophages were stimulated through TLR-3 for 24 h. Later, stimulated macrophages were harvested, washed, and then pulsed with dextran-FITC (1 mg/ml) for 2 h. Later, cells were washed extensively with ice-cold PBS-FBS-1% followed by fixation with paraformaldehyde (1×). Data were collected using BD FACS Aria flow cytometer and analyzed with BD DIVA software. Cells maintained at 4°C were used as control. For confocal analysis, cells pulsed with dextran-FITC (1 mg/ml) as described above were washed extensively (4×) with ice cold PBS (1×) and fixed with paraformaldehyde (4%). For imaging, cells were placed on poly-l-lysine coated cover slips and imaged using Zeiss confocal laser microscope (Nikon, Tokyo, Japan). *Z*-stacks were taken to exclude the interference of dextran bound to the surface. Results were examined by image analysis software.

### Tumor Model

C57BL/6 female mice were inoculated s.c. 5 × 10^5^ MC38 cells/mouse. On day 10 (when tumors were measurable or palpable), mice were administered (s.c.) TLR-3L (50 µg/mouse) and IFN-αβR (100 μg/mouse). Administration of TLR3-L was repeated three times with a gap of 3 days. Tumor growth was monitored every 2–3 days, in individually tagged mice by measuring two opposing diameters with a set of calipers. Tumors area was calculated as diameter1 × diameter2. Results are presented as the mean tumor size (squared millimeters) ± SD for every treatment group at various time points until the termination of the experiment.

### Immune Response in Tumor Model of Mice

Tumors were incised from TLR-3L-treated and control animals. For single cell preparation, tumor was chopped into small pieces and treated with collagenase D and suspended in RPMI 1640 supplemented with 10% FCS at 37°C for 30 min. Later, cells were passed through cells strainer (70 µm) followed by phenotypic assessment for the identification of different subsets of macrophages.

### RNA Isolation and cDNA Synthesis

Total RNA was extracted from fresh tumor and cells using RNeasy kit (Qiagen, Limburg, Netherlands), according to the manufacturer’s protocol. For cDNA synthesis, ~1 μg total RNA was reverse-transcribed into cDNA using Maxima first strand cDNA synthesis kit for quantitative RT-PCR (RT-qPCR) (Thermo Fisher Scientific, Waltham, MA, USA). cDNA was stored at −80°C for RT-qPCR.

### Western Blot Blotting for the Detection of iNOS

Polarized macrophages were stimulated with poly (I:C) as mentioned above. Later, cells were harvested, washed, and lysed in lysis buffer (RIPA buffer, protease, and phosphatase inhibitor cocktail). In SNs, proteins were estimated and equal concentration was subjected to SDS-PAGE. After transfer to nitrocellulose membrane and subsequent blocking, the membranes were immunoblotted with Abs against iNOs and actin as a loading control. Blots were developed using chemiluminescence kit (Amersham Pharmacia Biotech, Buckinghamshire, UK). Blots were scanned with the help of phosphoimager (Fujifilm, Tokyo, Japan), and image analysis was performed with MultiGuage software.

### T-Cell Help Experiment

Mice were sensitized by s.c. injection of OVA (100 µg). After 7 days, lymph nodes were isolated and single cell suspension was prepared to purify CD4 T cells using magnetic associated cells sorting. Purified CD4 T cells were labeled with efluor dye and cocultured with OVA-loaded macrophages at a ratio of 10:1 (T cells: Macrophages) for 5 days (10).

### THP-1 Macrophage Experiment

THP-1 cells were stimulated with PMA (160 nM) for 6 h for their differentiation into macrophages. Later, differentiated macrophages were washed and treated with IL-4 (20 ng/ml) for 24 h for their polarization toward M2 subtype. After 24 h, cells were washed and stimulated with poly (I:C) for 24 h.

### Human Blood Monocyte-Derived Macrophages

PBMCs were seeded in a tissue culture-treated Petri dish in RPMI media that was supplemented with 10% FBS, l-glutamine and penicillin–streptomycin. After 4 h, floating cells were removed and fresh 5 ml media having 50 ng/ml of recombinant hM-CSF was added. Every third day, 5 ml fresh media having 50 ng/ml rhM-CSF was added. Half medium was replaced at day 10 having 50 ng/ml rhM-CSF. On day 14, macrophages were harvested and treated with TGF-β (10 ng/ml) for 24 h for their polarization toward M2 subtype. After 24 h, cells were washed and stimulated with poly (I:C) for 24 h.

### *In Silico* Dataset for Analysis of TLR-3 and Type I Interferon Signaling Cascade and Its Correlation With Human Colon Adenocarcinoma (COAD) Patients

In Reactome pathway browser,^1^ we conducted a query search to obtain the downstream signaling events and the transcription factors unregulated post TLR-3 stimulation and type I interferon (IFN-αβ) in humans (11). To further explore the survival correlation in COAD patients, we used the OncoLnc tool^2^ to plot Kaplan–Meier graphs using the TCGA survival data of the COAD patient (12). These automatically generated Kaplan–Meier plots, with the log *p*-values, required the studied gene names and the values of lower and higher percentiles as input for the OncoLnc tool.

### Quantitative RT-PCR

Quantification of gene expression was performed using RT-qPCR analysis. The final reaction of RT-qPCR was performed in a volume of 10 µl, consisting of 1× SYBR green, 0.2 µM forward primer, 0.2 µM reverse primer, and 50–100 ng cDNA. Reactions were performed at 95°C for 15 s and 60°C for 30 s for 40 cycles in Applied Biosystems (Waltham, MA, USA) step one PCR. PCR program was set according to the manufacturer’s instructions. The mRNA expression unit of target gene against an internal control, GAPDH was calculated by ΔCT method. The difference (ΔCT) between the mean values in the duplicate samples of target gene and those of GAPDH were calculated by Microsoft Excel and the mRNA expression unit was expressed as 2^−(ΔCT*10,000).^

### Statistics

All statistical calculations were conducted using graph pad prism 5. For comparison between groups, statistical analysis was done by Student’s *t*-test. Comparison of survival curves was done by “log-rank (Mantel–Cox) Test.” *p*-Values < 0.05 was considered as significant.

## Results

### M1, M2a, and M2c Macrophages Have Distinct Phenotypes

**Figure 1:**
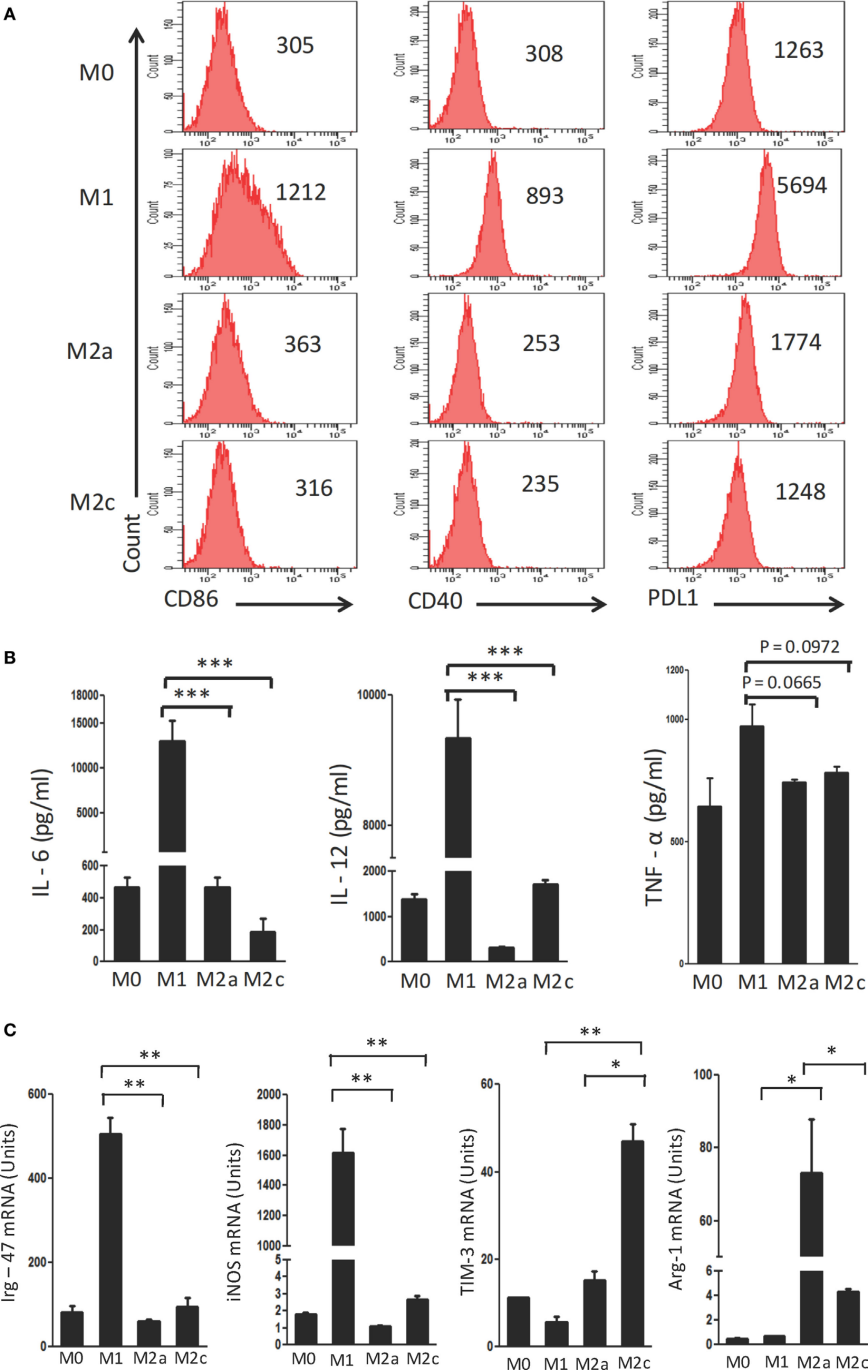
Polarization of macrophages to M1, M2a, and M2c phenotypes. Bone marrow cells (BMs) were cultured in the presence of conditioned medium containing M-CSF for 6 days. Later, macrophages were differentiated into M1, M2a, and M2c phenotypes using LPS + IFN-γ, IL-4 + IL-13, and TGF-β + IL-10, respectively. The M1, M2a, and M2c macrophages (F4/80^+^ gated cells) were characterized by the **(A)** display of CD86, CD40, and PDL-1 through flow cytometry. Number in the inset indicates the MFI; **(B)** secretion of IL-6, IL-12, and TNF-α estimated in the culture SNs by ELISA; **(C)** expression of *Irg-47, iNOs, Tim-3*, and *Arg-1* by quantitative RT-PCR. Data shown as mean ± SD are representative of two to three independent experiments (*p < 0.05; **p < 0.01; ***p < 0.001).

Macrophages were polarized in conditioned media for M1 (LPS + IFN-γ); M2a (IL-4 + IL-13) and M2c (TGF-β + IL-10) phenotypes. These cells were characterized on the basis of surface marker expression and release of cytokines. It was noticed that M1 macrophages showed upregulation but M2a and M2c displayed minor change in the expression of CD40, CD86 and PDL-1 by flow cytometry (Figure 1A). Further, augmented release of cytokines IL-6, IL-12, and TNF-α was observed in M1 macrophages by ELISA, as compared to the M0 subtype (Figure 1B). In contrast, M2a and M2c macrophages demonstrated significantly lower production of IL-6, IL-12, and TNF-α than those with an M1 phenotype. Furthermore, the M1, M2a, and M2c phenotypes were confirmed by studying the expression of *Irg-47, iNOS, Arg-1*, and *Tim-3* at the mRNA level using RT-qPCR (Figure 1C). Interestingly, M1 but not M2a and M2c macrophages showed significant elevation in the expression *Irg-47* and *iNOs* than M0; whereas M2a and M2c macrophages showed significant increase in *Arg-1* and *Tim-*3 expression, respectively.

### Signaling Delivered Through Innate Receptors Induces the Activation of M2a and M2c Macrophages

To find the most appropriate signal for reverting M2 macrophages to M1 subtype, macrophages were stimulated with the ligand of TLR-2, TLR-4, TLR-7, TLR-3, NOD-2, and CLRs. Interestingly, we observed that macrophages stimulated with poly (I:C) and LPS, which are ligands for TLR-3 and TLR-4, respectively, activated both M2a and M2c macrophages, as determined by the release of IL-6 (Figure S1A in Supplementary Material). However, M2c responded more efficiently to poly (I:C) triggering (Figure S1B in Supplementary Material). Therefore, we selected TLR-3 as a target for reversion of M2 Macrophages to M1 macrophages. Noteworthy, signaling delivered through TLR-3 using its ligand poly (I:C) showed dose-dependent increase in the IL-6 release by both M2a and M2c macrophages (Figures S1A,B in Supplementary Material). Since, optimum release of IL-6 was observed at a dose of 50 µg/ml of poly (I:C); thereby this concentration was selected for all the subsequent experiments.

### Triggering Through TLR-3 is Not Toxic for Macrophages

TLR-3L activated M0, M1, M2a, and M2c macrophages were incubated with PI and annexin V to stain the dead cells for assessing the toxic effect on activated macrophages. Importantly, no adverse effect was observed on the cells stimulated with TLR-3L (Figure S2 in Supplementary Material). Consequently, signifying that the dose of poly (I:C) selected for the stimulation of macrophages was not toxic.

### TLR-3 Triggering Reverts M2a and M2c Macrophages to M1 Subtype

**Figure 2:**
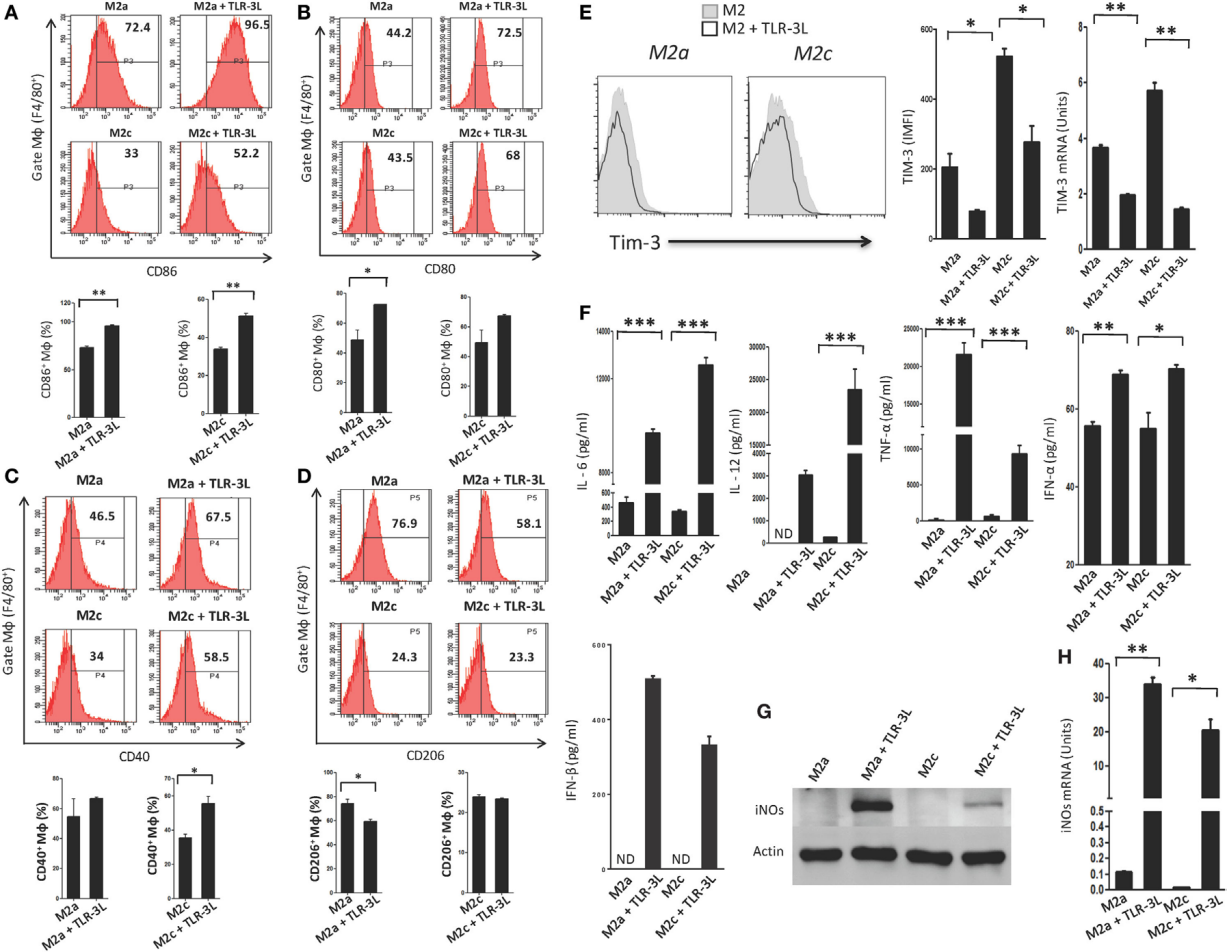
TLR-3 triggering reverts M2a and M2c macrophages to M1 phenotype. Macrophages were cultured in M2a and M2c differentiating conditions followed by treatment with TLR-3L for 24 h. The F4/80^+^ gated cells were assessed for the expression of **(A)** CD86; **(B)** CD80; **(C)** CD40; **(D)** CD206. Number in the inset of flow cytometry histogram indicates the percent of positive cells and expressed as bar diagram (lower panel). **(E)** The expression of Tim-3 was monitored by flow cytometry. The results are confirmed by quantitative RT-PCR (RT-qPCR) and the data depicted as bar diagram (side panel). **(F)** The IL-6, IL-12, TNF-α, IFN-α, and IFN-β were quantified in the culture SNs by ELISA. **(G)** The iNOs was detected in the whole cells lysate by western blotting and further confirmed by **(H)** RT-qPCR (side panel). The data expressed as mean ± SD are representative of two to three independent experiments (*p < 0.05; **p < 0.01; ***p < 0.001).

We observed that signaling delivered through TLR-3 considerably augmented the expression of CD86, CD80, and CD40 on M2a and M2c macrophages, which are the established markers for the M1 subtype (Figures 2A–C). Simultaneously, a significant decrease in the expression of CD206 was noted on M2a macrophages, a specific marker for alternatively activated macrophages (Figure 2D). However, no change was observed in M2c macrophages. Co-inhibitory molecules such as Tim-3 play a critical role in the negative regulation of T cell responses in lymphoid organs and peripheral non-lymphoid tissues to control immune responses and inflammation (13). Therefore, we checked the expression of Tim-3 on TLR-3-stimulated macrophages. Interestingly, we noticed a significant decrease in the expression of TIM-3 on the TLR-3 reverted M2a and M2c macrophages by flow cytometry. Additionally, change in the levels of *Tim-3* was further substantiated at the mRNA level by RT-qPCR (Figure 2E). These results illustrated that TLR-3 signaling in M2a and M2c macrophages skewed their phenotype toward M1-like macrophages through the induction of the activation markers and suppression of co-inhibitory receptors. We also noted significant release of pro-inflammatory cytokines IL-6, IL-12, TNF-α, IFN-α, and IFN-β than the control cells (Figure 2F). It is notable that TLR-3 signaling of M2a and M2c macrophages substantially upregulated the expression of PDL-1 and enhanced the secretion of IL-10 (Figures S3A and S4A in Supplementary Material), which is necessary to control the inflammatory response. TLR-3 efficiently reverted the M2a and M2c macrophages to an M1 subtype. Therefore, next, we assessed whether signaling through TLR-3 could inhibit the Polarization of macrophages to M2a and M2c phenotypes. Macrophages supplemented with M2a (IL-4 + IL-13) and M2c (TGF-β + IL-10) polarizing conditions were cultured in the presence of TLR-3L for 24 h. Interestingly, TLR-3 signaling blocked M2a and M2c polarization, as revealed by the upregulation of the costimulatory molecule CD86 and the downregulation of TIM-3 and CD206 (Figures S6A–C in Supplementary Material).

Furthermore, the status of reverted macrophages was confirmed by examining the exhibition of iNOs by western blotting (Figure 2G). Interestingly, TLR-3-stimulated M2a and M2c macrophages showed higher levels of iNOs than the control cells. It is important to mention that M2a macrophages responded more efficiently to TLR-3L than M2c macrophages. The induction of *iNOs* was further confirmed at the mRNA level by RT-qPCR (Figure 2H). Overall, the results confirm the reversion of M2a and M2c macrophages to M1 phenotype by TLR-3 signaling, as demonstrated through change in the phenotypic markers by using four distinct methods, *viz*, flow cytometry, western blotting, ELISA, and RT-qPCR.

### Stimulation Through TLR-3 Enhances the Antigen Uptake Ability of M2a Macrophages and Subsequently Their Capacity to Activate T Cells

**Figure 3:**
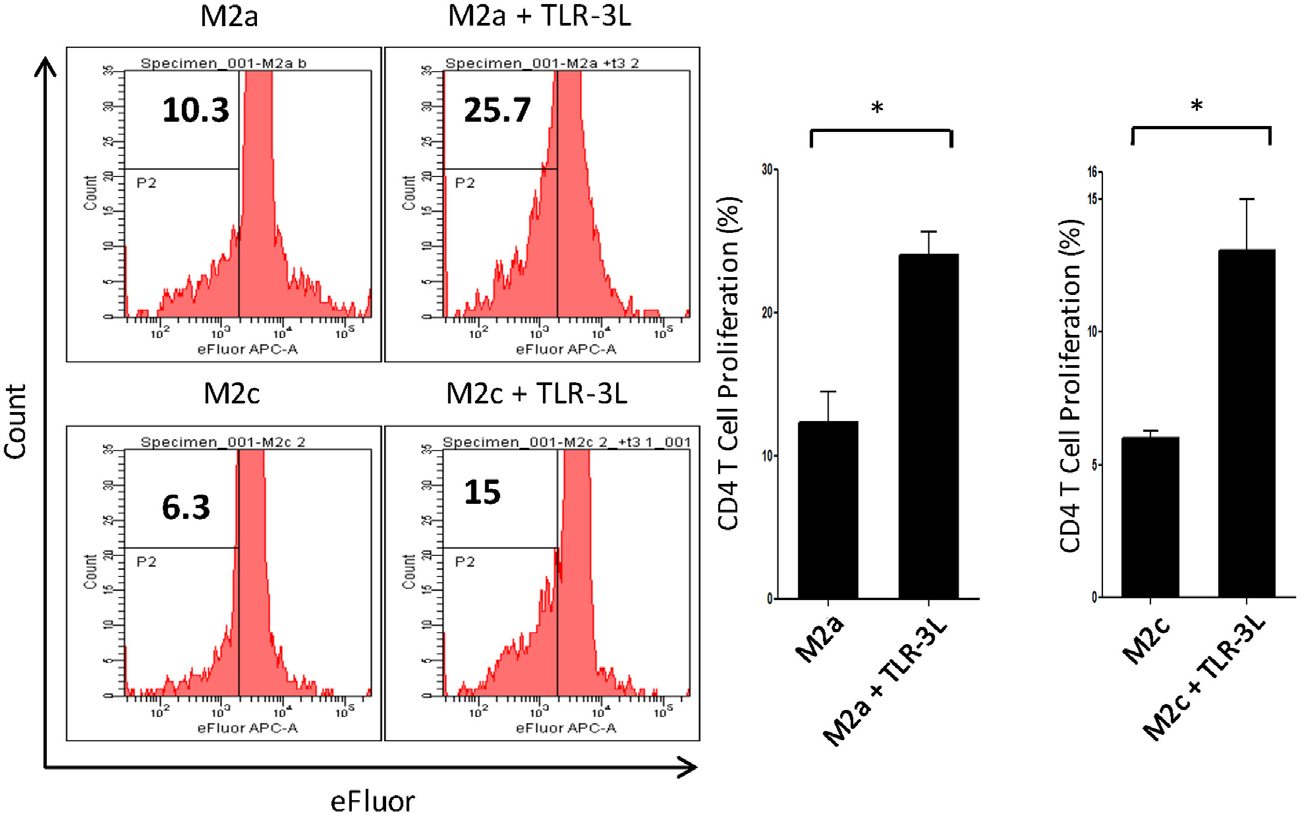
Signaling through TLR-3 increases macrophages capacity to activate CD4 T cells. M2a and M2c macrophages loaded with antigen were cocultured with efluor-labeled antigen-specific CD4 T cells isolated from lymph nodes of OVA immunized mice. The proliferation was assessed by eFluor-dye dilution assay. Number in the flow cytometry histogram indicates the CD4 gated efluor^lo^ cells. The results are also illustrated as bar graphs (side panel). The data expressed as mean ± SD are representative of two independent experiments (*p < 0.05).

M2a macrophages were stimulated with TLR-3L for 24 h and then assessed for their ability to uptake antigen. TLR-3 stimulation increased the phagocytosis of dextran-FITC as assessed by flow cytometry (Figure S5A in Supplementary Material). These results were further validated by confocal microscopy experiments (Figure S5B in Supplementary Material). Consequently, we determined the potential of TLR-3 stimulated M2a and M2c macrophages to activate CD4 T cells. Intriguingly, TLR-3 stimulation substantially increased the capacity of both M2a and M2c macrophages to induce the proliferation of CD4 T cells (Figure 3). Although, M2a and M2c macrophages are considered as weak antigen presenting cells compared to M1 phenotype, but stimulation through TLR-3 could noticeably improve the effectiveness of antigen presentation.

### Regression of Tumor Growth in Mice upon TLR-3L Administration is IFN-αβ Signaling Dependent

**Figure 4:**
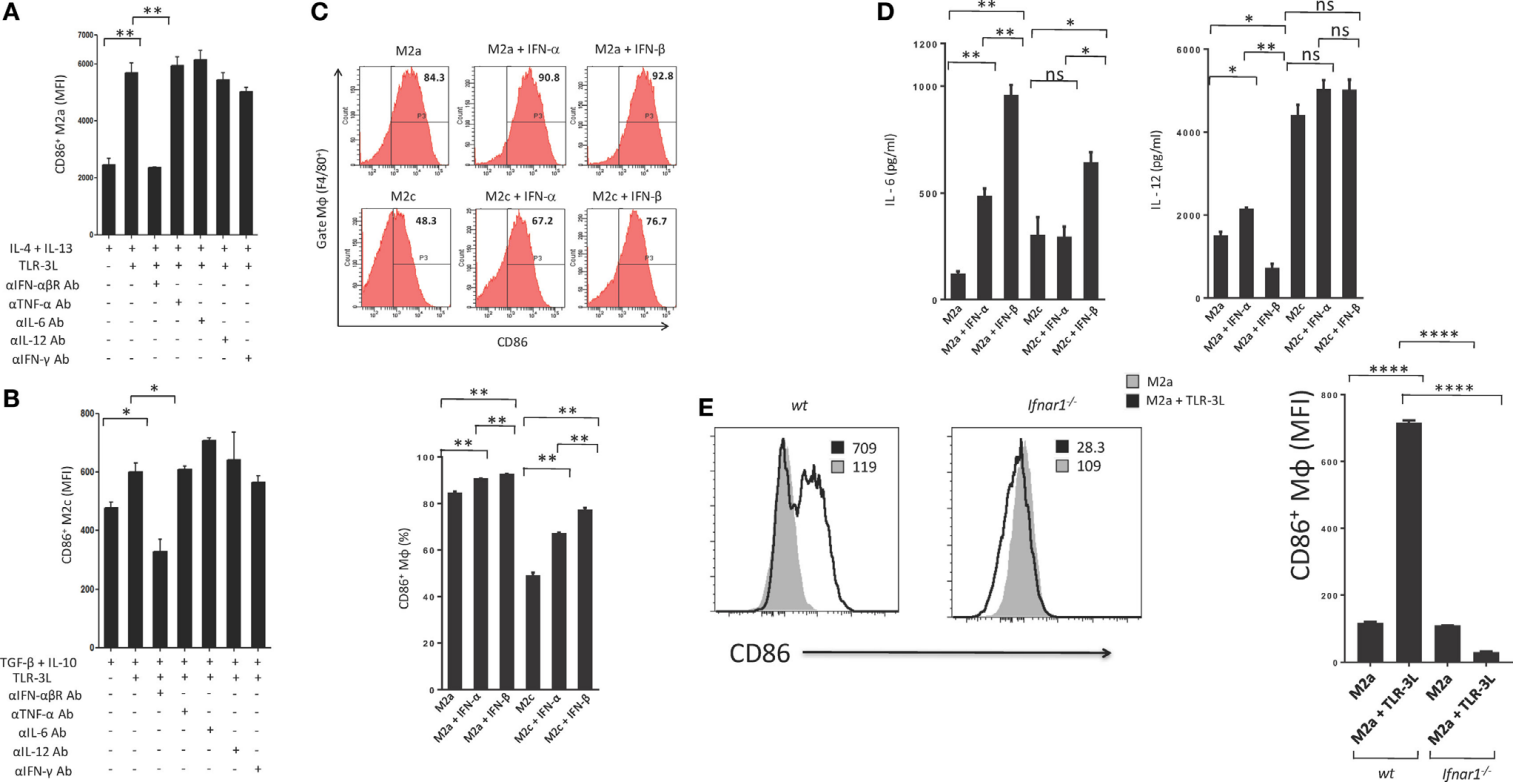
IFN-αβ released during TLR-3 triggering reverts the phenotype of macrophages from M2 to M1 subtype. Polarized macrophages **(A)** M2a; **(B)** M2c were treated with TLR-3L in the presence of Abs to IFN-αβR, TNF-α, IL-6, IL-12, and IFN-γ for 24 h. The expression of CD86 was studied on F4/80^+^ positive cells. **(C)** M2a and M2c macrophages were treated with IFN-α and IFN-β for 24 h. The F4/80^+^ gated cells were assessed for the expression of CD86. Number in the inset of flow cytometry histogram indicates the percent of positive cells, and the data are also expressed as bar diagram (lower panel). The secretion of cytokines was quantified in the culture SNs for **(D)** IL-6 and IL-12 by ELISA **(E)** Bone marrow cells (BMs) obtained from WT and Ifnar1^−/−^ mice were cultured with conditioned medium supplemented with M-CSF for 6 days. Later, macrophages were differentiated into M2a phenotype using IL-4 + IL-13 and followed by the treatment with TLR-3L for 24 h. The F4/80^+^ gated cells were assessed for the expression of CD86. Number in the inset of flow cytometry histogram indicates the MFI. Consecutively, the data are also expressed as bar diagram (right side panel). The data expressed as mean ± SD are representative of two independent experiments (*p < 0.05; **p < 0.01; ****p < 0.0001).

Type I IFNs are rapidly produced by many cell types in response to immune and/or inflammatory stimuli. Poly (I:C) was previously shown to stimulate mouse conventional DCs, bone morrow-derived DCs and macrophages to produce type I IFN (14, 15). Therefore, we asked whether the mechanism required to induce the polarization of M2a and M2c macrophages toward M1 is IFN-αβ dependent. We cultured TLR-3L-treated M2a and M2c macrophages in the presence of anti-IFN-αβR blocking antibody. It was noticed that blocking of IFN-αβR failed to revert macrophages M2a and M2c to M1 phenotype (Figures 4A,B). We demonstrated the specificity of IFN-αβ by neutralizing the activity of cytokines including IL-6, IL-12, TNF-α, and IFN-γ by their respective antibodies. Unlike blocking IFN-αβR, we observed no change in the activation of macrophages by the neutralization of the function of TNF-α, IL-6, IL-12, and IFN-γ. Similar results were observed with M2c macrophages. Further, to validate our results we stimulated M2a and M2c macrophages with recombinant IFN-α and IFN-β. We observed significant upregulation of the expression of the costimulatory molecule CD86, confirming the reversion of M2a and M2c macrophages toward M1 subtype (Figure 4C). Further, we observed that IFN-α- and IFN-β-treated M2 macrophages showed enhanced secretion of IL-6 and IL-12 (Figure 4D). To establish the specificity of IFN-αβ signaling, we compared the efficacy of BMDM from WT and *Ifnar1^−/−^* mice. Macrophages obtained from WT were able to revert to M1 phenotype when treated with TLR-3L. In contrast, macrophages derived from *Ifnar1^−/−^* animals exhibited no enhancement in the display of CD86 (Figure 4E). These data demonstrate that IFN-αβ signaling is required for the reversion from M2 to M1 polarization state of the BMDM.

**Figure 5:**
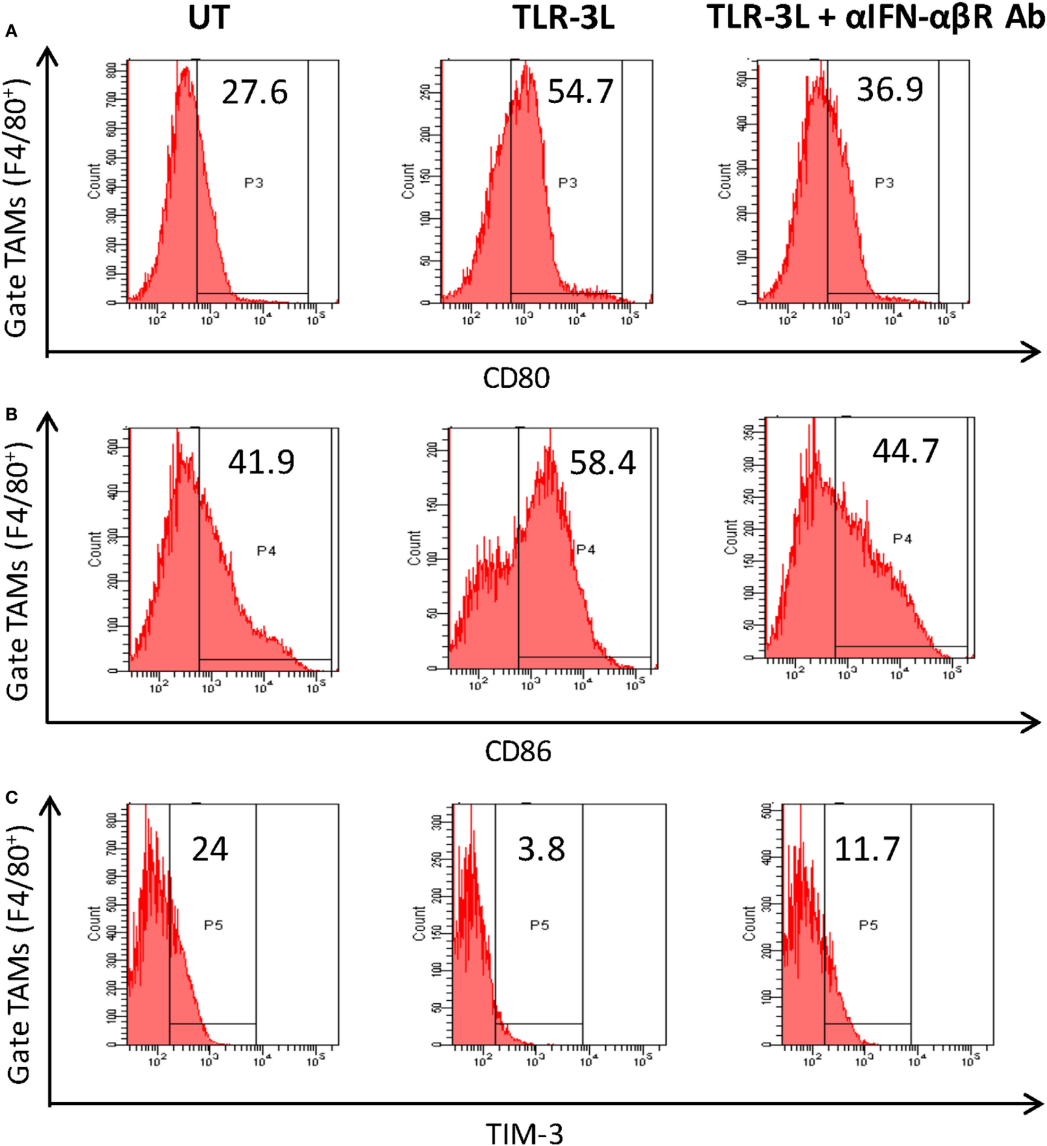

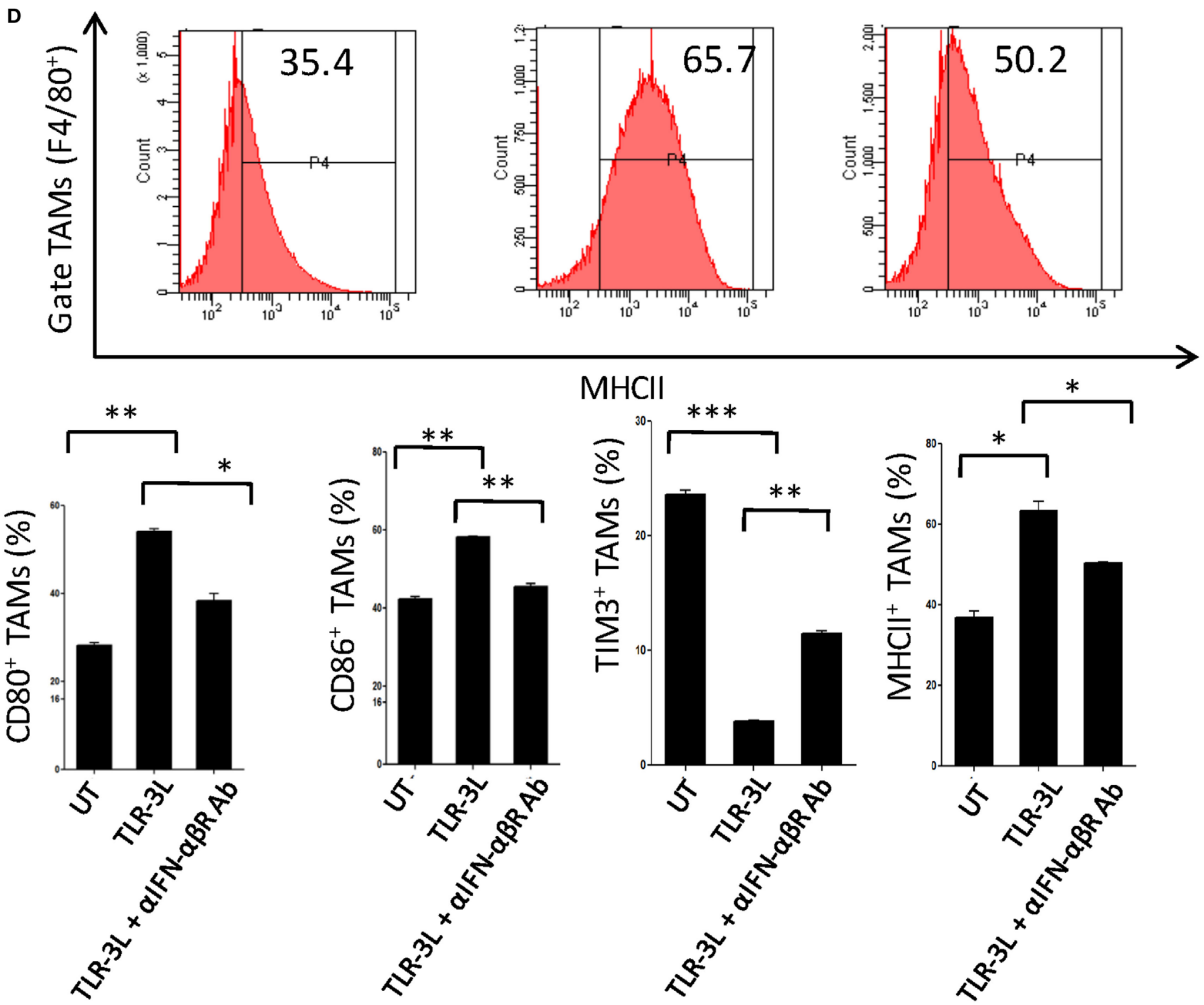
Administration of TLR-3L reverts the M2 phenotype of tumor-associated macrophages to M1 subtype. TLR3-L was administered in tumor-bearing mice. After 21 days, cells were isolated from the excised tumors and tumor-associated macrophages (TAMs) were assessed by flow cytometry for the expression of **(A)** CD80; **(B)** CD86; **(C)** TIM-3; and **(D)** MHC-II. The bar graphs expressed as mean ± SD represents the percentage change in the phenotypic markers on the F4/80^+^ positive cells (lower panel). The data shown are representative of two independent experiments (*p < 0.05; **p < 0.01; ***p < 0.001).

To determine the role of IFN-αβ signaling in tumor-associated macrophage (TAMs), we used anti-IFN-αβ antibodies to inhibit this signaling pathway *in vivo*. We observed that TLR-3 stimulation efficiently augmented the level of activation markers on TAMs, as observed by the upregulation of CD80, CD86, and MHC-II (Figures 5A–C). Simultaneously, decrease in the display of Tim-3, a marker associated with suppressive phenotype was noticed (Figure 5D). Interestingly, when anti-IFN-αβ antibodies were administered in TLR-3L-treated animals; there was downregulation in the expression of MHC-II, CD80, and CD86 molecules and increase in the exhibition of Tim-3 in TAMs. Thus, categorically establishing the contribution of IFN-αβ signaling in TLR-3-mediated reversion of TAMs. TAMs constitute a major part of the infiltrated cells in human cancers. It has been reported that the tumor-infiltrating macrophages have been associated with poor prognosis of the disease (16). Therefore, this study demonstrates a crucial role of TLR-3 stimulation in suppressing the pro-tumorigenic functions of TAMs.

### Administration of TLR-3L in the Animals Regress the Tumor Growth

**Figure 6:**
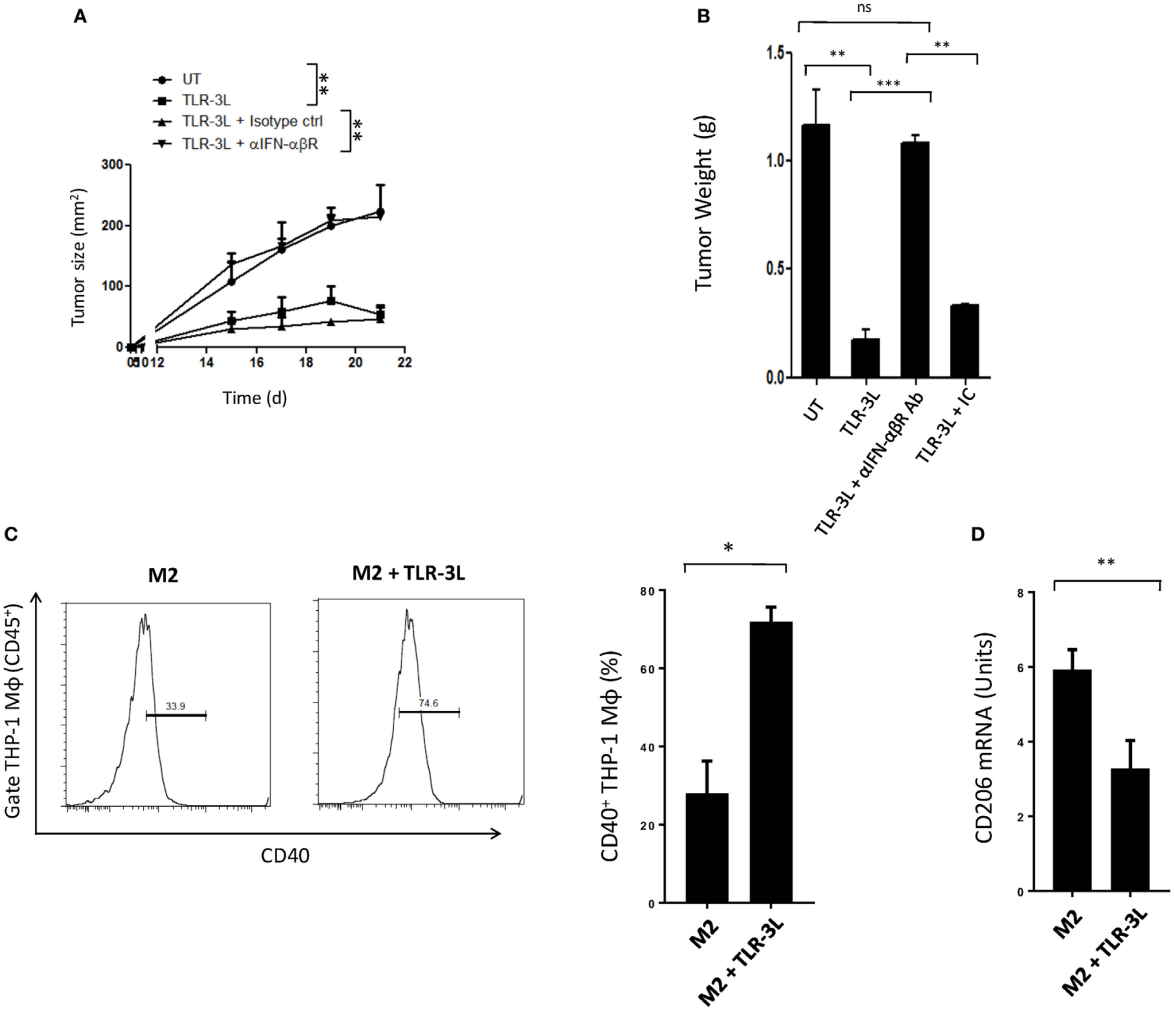
The mechanism involved in the regression in the tumor size in animals administered TLR-3L requires IFN-αβ signaling. **(A)** Tumor-bearing mice were injected s.c. with three doses of TLR-3L with a gap of 3 days. The size of the tumor is indicated in squared millimeter at different time points. **(B)** Bar graph represents weight of the tumors in grams. The specificity of the involvement of IFN-αβR was established through blocking Abs against IFN-αβR. Data shown as mean ± SD are representative of two independent experiments. Abbreviations: IC, isotype control; ns, non-significant. **p < 0.01; ***p < 0.001. TLR-3 triggering reverts human M2 macrophages to M1 phenotype. THP-1 macrophages were cultured in M2 differentiating conditions followed by treatment with TLR-3L for 24 h. The CD45^+^-gated cells were assessed for the expression of **(C)** CD40. Number in the inset of flow cytometry histogram indicates the percent of positive cells. The data are also expressed as bar diagram (right panel). **(D)** The expression of CD206 was monitored by quantitative RT-PCR and depicted as bar diagram. The data expressed as mean ± SD are representative of two independent experiments (*p < 0.05; **p < 0.01).

Tumor-bearing mice were injected with TLR-3L three times with a gap of 3 days. The effectiveness of TLR-3L was evaluated after 10 days of s.c. inoculation of murine cancer cell line MC38. As compared to control, the animals treated with TLR-3L exhibited significant reduction in the tumor growth (Figure 6A; Figure S7A in Supplementary Material). Further, a decrease in the weight of tumor was observed in the TLR-3L-treated animals (Figure 6B). We next asked whether the reversion of macrophages is operating *in vivo* through IFN-αβ signaling. Interestingly, the cohort injected with anti-IFN-αβ blocking antibodies showed significant progression of tumor, as compared to TLR-3L-treated animals (Figure 6A; Figure S7A in Supplementary Material). No change was observed in the tumor in the control mice receiving isotype-matched antibodies. Hence, Data suggest that for *in vivo* reversion of TAMs, IFN-αβ signaling is critical and utilizing TLR-3L may be an effective antitumor therapeutic approach.

### TLR-3 Triggering Reverts Human M2 Macrophages to M1 Phenotype

Finally, we investigated that the reversion of murine M2 macrophages to M1 phenotype, can be replicated with human macrophages. Signaling delivered through TLR-3 substantially augmented the expression of CD40 on M2 macrophages differentiated from human THP-1 cells. CD40 is an established marker associated with M1 macrophages (Figure 6C). In contrast, a significant decrease in the expression of *CD206* was noted on M2 macrophages. CD206 is a marker for M2 macrophages (Figure 6D). To further validate our results, we used primary human macrophages to investigate reversion of M2 macrophages to M1 macrophages. Stimulation given through TLR-3 significantly decreased the expression of *CD163* in M2 macrophages. CD163 is a marker for M2 macrophages (Figure S8 in Supplementary Material). Reversion of human primary and THP1 macrophages authenticated our findings noticed with murine mouse macrophages. Further, these findings indicate a clinical relevance of TLR-3 signaling and its potential for therapeutic applications against cancer by targeting TAMs.

### TLR-3 Signaling Cascade Leads to Activation of Type I Interferon Transcription Factors, Which Subsequently Induces Effective Innate Immune Response

In the final part of the present study, we decided to explore the TLR-3 and IFN-αβ signaling nexus in COAD patients. First to test the hypothesis of TLR-3 ligand stimulation leads to upregulation of type I interferon and its immune response in humans, we analyzed the signaling cascade events *via* reactome pathways database (11). Visual representation of the signaling event and interacting macromolecules is represented in Figure S9 in Supplementary Material. Interferon regulatory factor 7 and 3 (IRF7, IRF 3) are the two essential transcriptional regulators of type I interferon; IFN-α/β, whose phosphorylation, dimerization, and translocation to nucleus leads to the binding to interferon-stimulated response element in the promoter region; thus leading to the transcription of IFN-α/β (17–19) (Figure S9A in Supplementary Material). Further, we explored IFN-α/β-dependent immune responses induced by stimulation of these type I cytokine on a cell and their downstream IFN-stimulated genes. Translated proteins induce a robust immune response marked by tumoricidal and inflammatory markers, such as MHC class I, OAS proteins, ADAR, PSMB8, and XFA1 (20–22) (Figure S9B in Supplementary Material). Notably, formation of IRF7 and IRF3 post TLR-3 ligand stimulation, and subsequent IFN-α/β transcription indicates release of these cytokines (23–25).

Finally, using OncoLnc, we assessed the clinical significance of the signature genes for M1, M2, and TAMs in COAD patients obtained from TGCA recourse portal Figure S10 in Supplementary Material (12, 26). Important highlights in the Kaplan–Meier survival plots may be concluded as patients with higher expression in M1 genes, cytokines and pro-inflammatory markers, such as TDRD7, IL-1β, CCL11, NOS2, TBK1, IFI-27, and PSMB8, exhibit significant survival prospect, as compared the groups with lower expression (Figure S10A in Supplementary Material). Critically, survival probability in COAD patients expressing elevated M2 and TAM related genes, *viz*, TGFβ1, CD68, FABP4, and VEGFA demonstrated lesser prospect of survival (Figure S10B in Supplementary Material) (27–30). In essence, an empiricist conclusion highlights the clinical relevance in reverting back pro-tumorigenic M2 macrophages back to their pro-inflammatory phenotype, where in stimulation of TLR-3 ligands may provide a novel therapeutic intervention for COAD patients.

## Discussion

Tumor progression is dependent on numerous mechanisms ensued by the tumors to prevent and suppress the cascade of antitumor events (31). Understanding of these mechanisms may provide the basis for the development of various immunotherapeutic approaches. Polarization of macrophages to M2 subtype is one of the well-evident facts responsible for the tumor progression (32, 33). Hence, exploration of the strategies targeting innate immunity molecules to skew M2 macrophages to M1 phenotype could be one of the effective strategies to explore against cancer.

Innate immunity has been targeted in tumor-bearing animal models with promising results (34). Treatment with TLRs agonist is a commonly used procedure that results in rapid activation of innate and adaptive immunity. The most commonly used TLR agonists are cytosine-phosphorothioate guanine oligonucleotides for TLR-9, imiquimod for TLR-7, and poly (I:C) for TLR-3 (7, 35, 36). Although, signaling through TLR-3 showed a promising effect on the regression of tumor growth, nothing has been elucidated on its influence on TAMs. M1 macrophages are known to play a fundamental role in the resistance to tumor progression (37). In contrast, M2 phenotype is responsible for the progression of tumor (38, 39). Consequently, our study focused on defining the role of TLR-3 signaling in reverting the M2 macrophages to M1 phenotype. Stimulation of TLR-3 oriented macrophages to an M1 phenotype, as evidenced by (i) upregulation in the expression of costimulatory molecules; (ii) inhibition of co-inhibitory receptors; (iii) induction of IL-6, IL-12, TNF-α, iNOs; (iv) enhancement in antigen uptake; (v) improvement in the ability to prime T cells; (vi) blocking the polarization of macrophages toward M2a and M2c subtype; and (vii) significant increase *in vivo* of M1 macrophages and regression in tumor growth.

T cells play a critical role in the controlling and eliminating of cancer cells. M1 and M2 macrophages can regulate the activation and suppression of T cells, respectively. Therefore, it is important to regulate the differentiation and activation of macrophage subtypes that can activate T cells. It is important to mention that TLR-3 signaling augmented the expression of costimulatory molecules CD80, CD86, CD40 on macrophages and their production of cytokines such as IL-6, IL-12, and TNF-α. In contrast, downregulation of the inhibitory molecule Tim-3 was observed. This suggests that TLR-3 stimulation skews the macrophages toward M1 phenotype which not only activates T cells but also prevents tumor progression. Antitumor immunity is known to be suppressed through Tim-3 (40, 41). Recent studies in human and animal cancer models showed substantial role of Tim-3 in CD8 T cell exhaustion. Expression of Tim-3 on dendritic cells resulted in impaired response to nucleic acid-stimulated tumor immunity (42). Tim-3 is involved in the development of tumor-promoting M2 macrophages in colon cancer (43). To the best of our knowledge, this is the first report demonstrating that inhibitory molecule Tim-3 is highly expressed specifically on M2c macrophages. Furthermore, this finding suggests that signaling of M2 macrophages through Tim-3 might have an important function on the progression of tumor. However, this area needs comprehensive investigation. Importantly, we noted that TLR-3L treated macrophages inhibits the expression of TIM-3; thereby suggesting a therapeutic potential of targeting the TIM-3 mediated suppression of M2c macrophages. The information further substantiates the role of TLR-3 in restoring the antitumorigenic function of M2a and M2c macrophages.

Type-1 interferons are important cytokines in inducing protection against various diseases. They perform their function by acting directly on target cells or by activating immune cells (44, 45). Recent studies have very well defined the role of type-1 interferons in antitumor immunity. Our data also support the role of type-1 interferons in inducing antitumor immunity (45, 46). This study reveals a novel signaling axis by which a reduction in the density of tumor-associated M2 macrophages can slow the tumor growth. Additionally, it deciphers the importance of signaling through TLR-3 in effectively skewing “tumor-associated macrophages-M2” to “tumor-protective macrophage subtype-M1” *in vitro*, as well as *in vivo*, in the tumor microenvironment of the mice. Further, administration of TLR-3L in the animals reverted M2 macrophages to M1 macrophages through the involvement of IFN-αβ signaling and substantially arrested the growth of tumor. Furthermore, our study supports the paradigm, whereby a conversion of tumor-associated M2 macrophages to M1 in the tumor microenvironment controls tumor growth.

## Ethics Statement

All experiments were approved by the Institutional Animal Ethics Committee of CSIR-IMTECH and performed according to the National Regulatory Guidelines issued by Committee for the Purpose of Supervision of Experiments on Animals (No. 55/1999/CPCSEA), Ministry of Environment and forest, Govt. of India. Animal experiments (C57BL/6 wild-type and ifnar^−/−^) were performed in accordance with the guidelines of Yale University’s Institutional Animal Care and Use Committee (Protocol No. 11264).

## Author Contributions

JA, AV, NK, and MT conceived the project. AV, NK, TA, SN, DD, MA, and DC performed *in vitro* experiments. AV, NK, and TA performed *in vivo* experiments. AV, NK, JA, DC, and OC analyzed data. AV, JA, and NK wrote the manuscript.

## Conflict of Interest Statement

The authors declare that the research was conducted in the absence of any commercial or financial relationships that could be construed as a potential conflict of interest.

## Footnotes

^1^https://reactome.org/PathwayBrowser/ (Accessed: June 8, 2018).

^2^www.oncolnc.org (Accessed: June 8, 2018).
