# Supplementary material for: TLR-3 Stimulation Skews M2 Macrophages to M1 Through IFN-αβ Signaling and Restricts Tumor Progression
Source: Front Immunol. 2018 Jul 19;9:1650. doi: 10.3389/fimmu.2018.01650 (PMC6060442; doi:10.3389/fimmu.2018.01650)
Supplement: Supplementary file 1 [file Presentation_1.PDF]

SUPPLEMENTARY MATERIAL

Figure S1

[A]

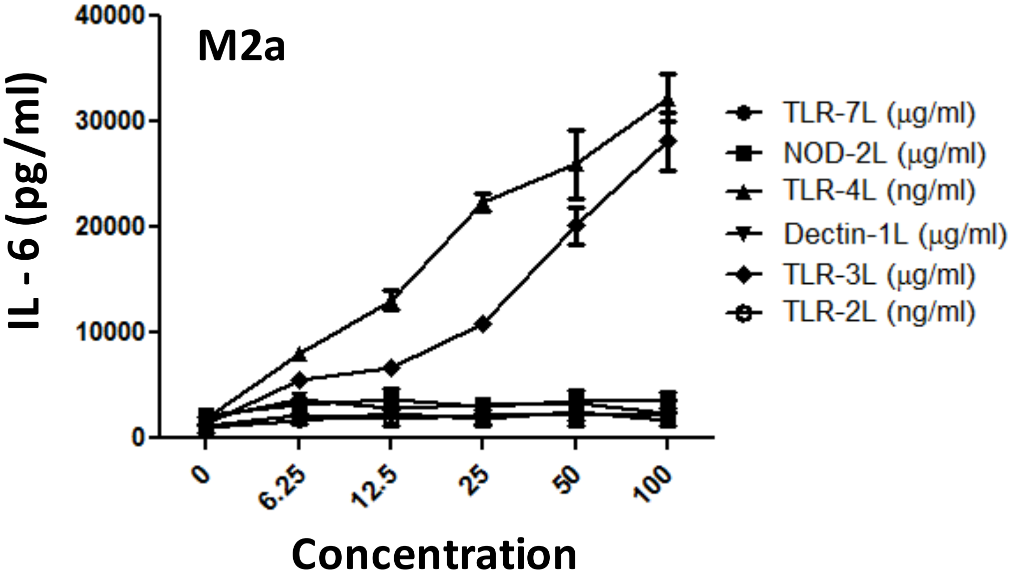

[B]

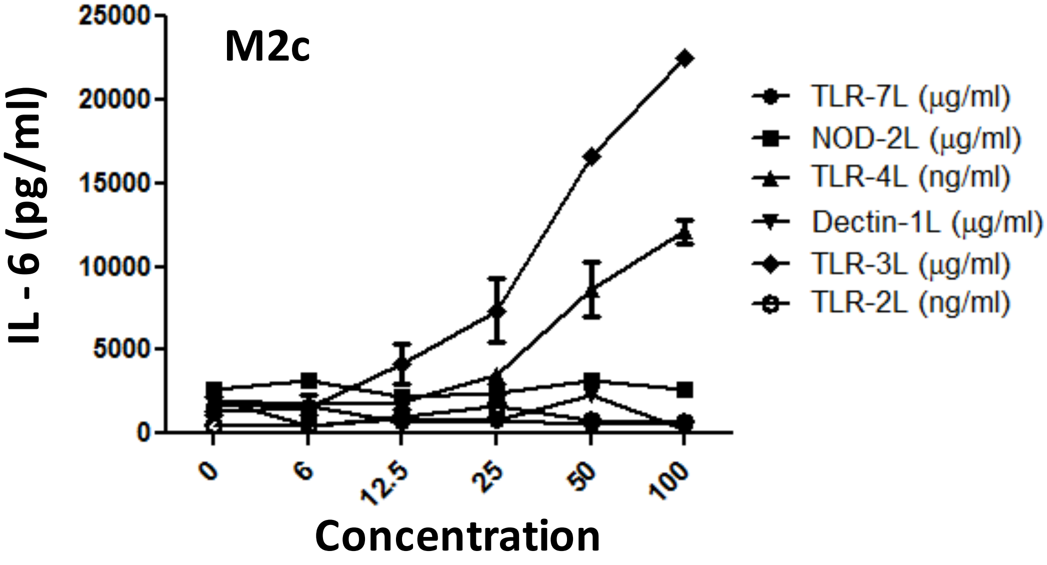

Figure S1. Triggering of M2a and M2c macrophages through TLR-3 augments the release of IL-6. Macrophages were cultured in the presence or absence of M2a and M2c polarization conditions, as mentioned in the legend to Figure 1. Later, cells were stimulated through TLR-2, TLR-3, TLR-4, TLR-7, dectin-1 and NOD-2 for 24h. Secretion of IL-6 by (A) M2a; (B) M2c was quantified in the culture SNs by ELISA. Data expressed as mean±SD (pg/ml) are representative of 2 independent experiments. Each assay was conducted in triplicate wells.

Figure S2

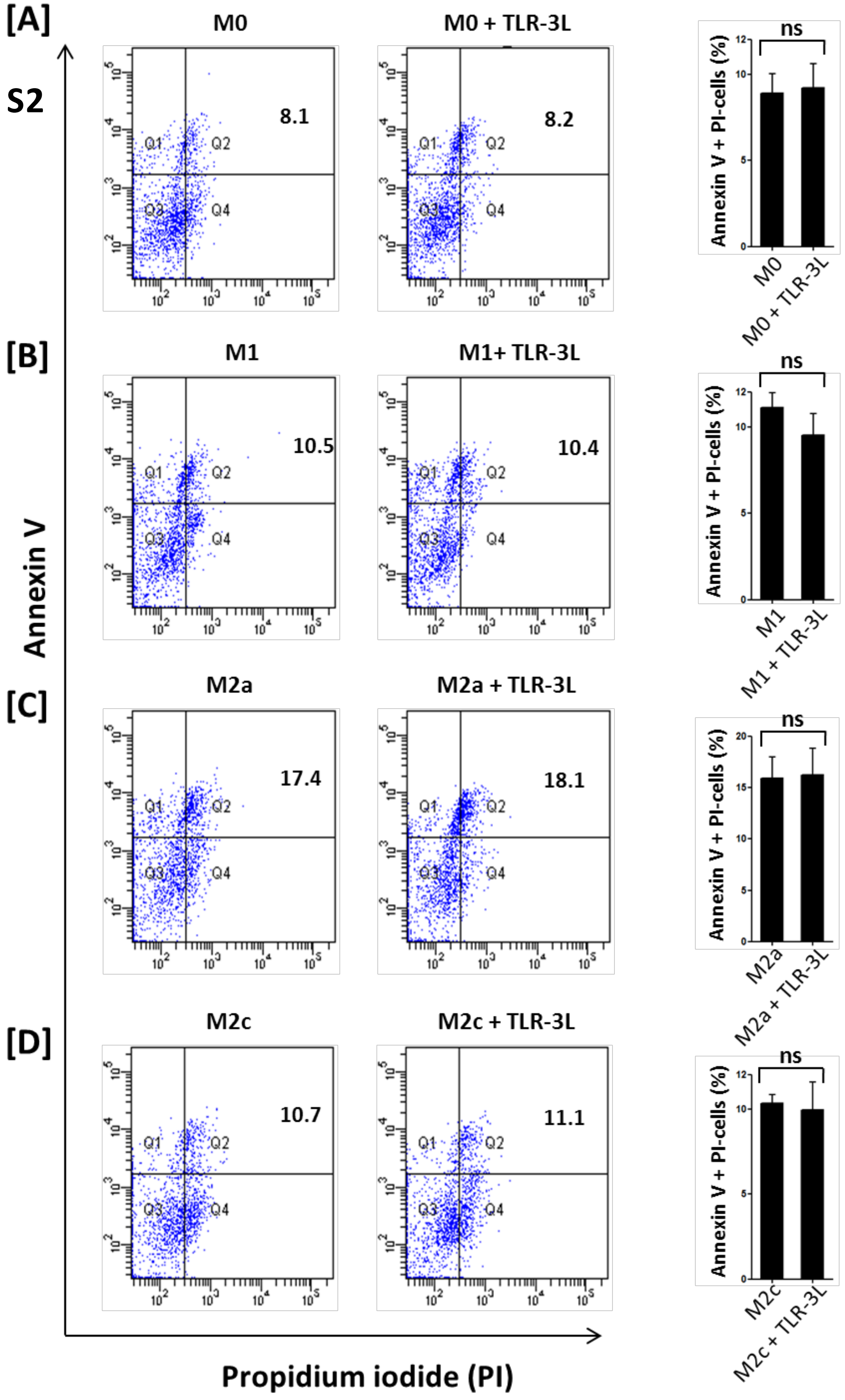

Figure S2. Signaling delivered through TLR-3 showed no effect on the viability of cells. Macrophages (A) M0; (B) M1; (C) M2a; (D) M2c stimulated with TLR-3L were incubated with propidium iodide and annexin V to stain dead or apoptotic cells, respectively. Number in the quadrant depicts the percentage of double positive cells for annexin V and PI. Data shown as mean±SD are representative of 2 independent experiments. ns: non significant.

Figure S3

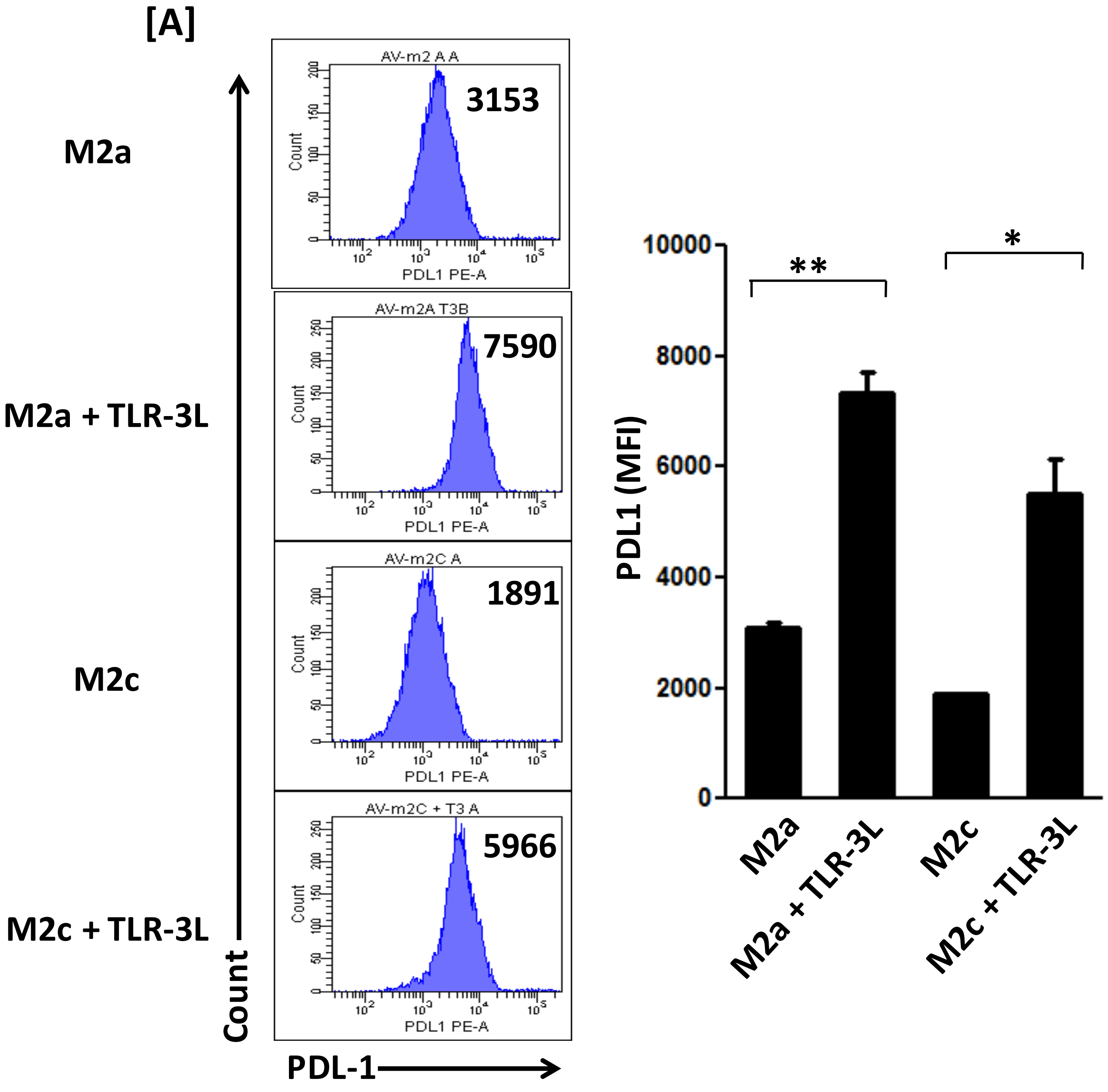

Figure S3. TLR-3 triggering reverted M2a and M2c macrophages to M1 phenotype. Macrophages were cultured in M2a and M2c differentiating conditions followed by treatment with TLR-3L for 24h. M2a and M2c cells (F4/80 gated population) were assessed for the expression of coinhibitory receptor (A) PDL-1. Number in the inset of the histogram indicates the MFI. Bar graph depicts change in MFI (side panel). Data represented as mean±SD are of 2 independent experiments. *p<0.05;**p<0.01.

Figure S4

[A]

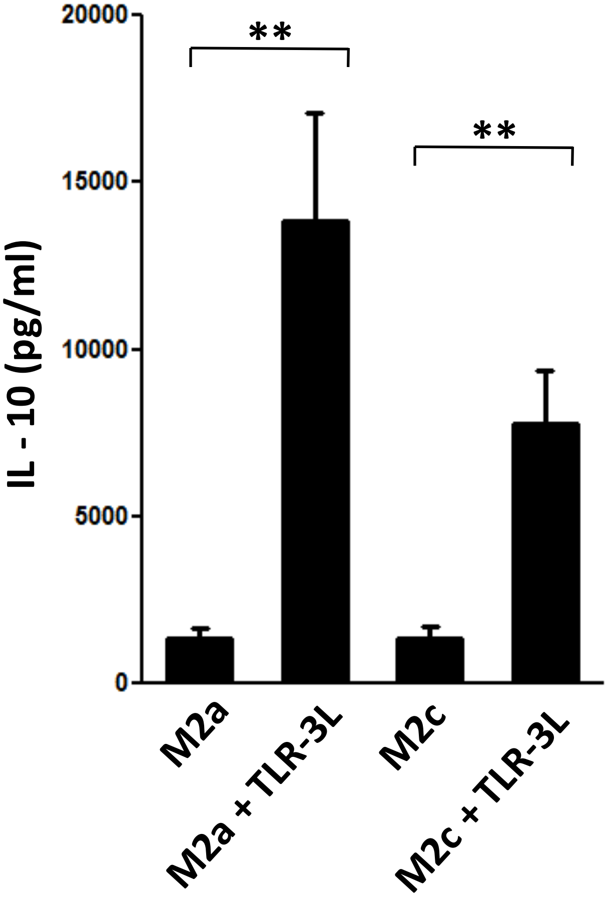

Figure S4. TLR-3 triggering of M2a and M2c macrophages induced the secretion of IL-10. Macrophages were cultured in M2a and M2c differentiating conditions followed by treatment with TLR-3L for 24h. The release of IL-10 was quantified in culture SNs by ELISA. The data expressed as mean±SD are representative of 3 independent experiments.**p<0.01.

Figure S5

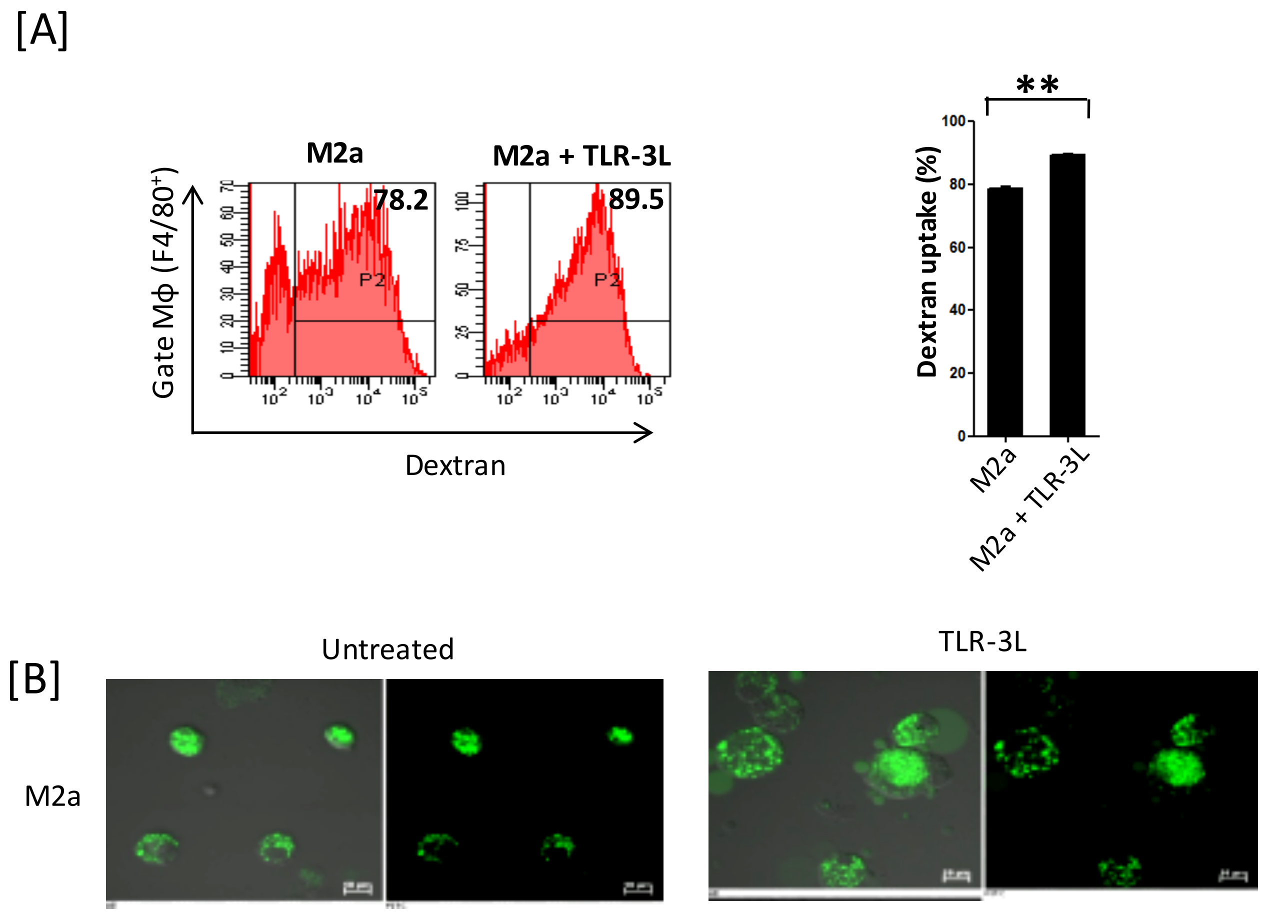

Figure S5. Signaling through TLR-3 augmented the antigen uptake ability of M2 macrophages. M2a macrophages were treated with TLR-3L for 24h. Later, antigen (dextran-FITC) uptake was assessed through (A) flowcytometry; (B) confocal microscopy at a magnification of 60x. Number in histogram depicts percentage of dextran FITC^{+} cells. Bar graph represents the percent of dextran-FITC^{+} cells. The data expressed as mean±SD are representative of 2 independent experiments. $^{**}p<0.01$.

Figure S6

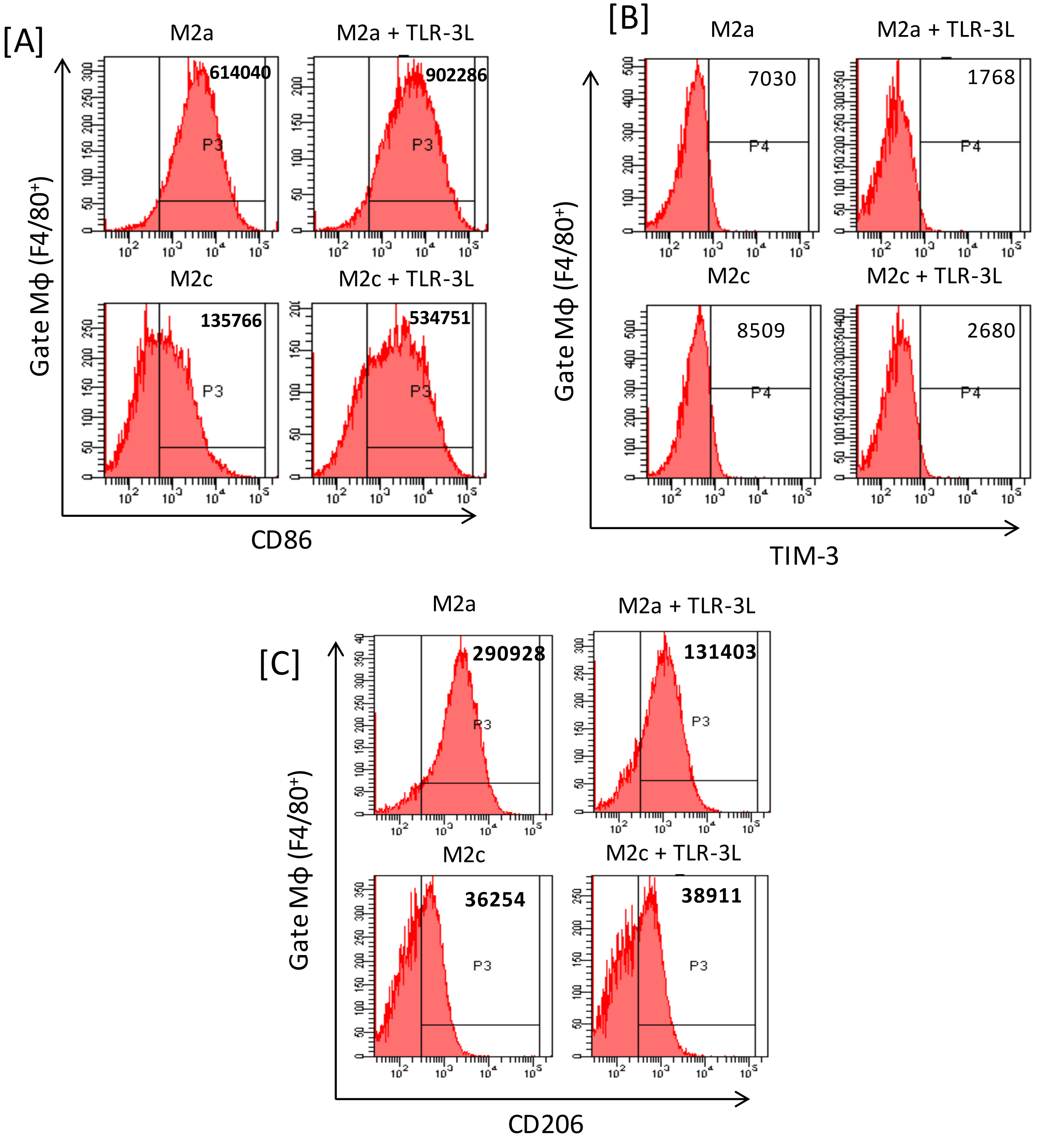

Figure S6. TLR-3 triggering prevented the polarization of M2a and M2c macrophages. Macrophages were incubated with TLR-3L during their polarization to M2a and M2c subtypes. Phenotypes of macrophages were monitored through the expression of (A) CD86; (B) TIM-3; (C) CD206 by flow-cytometry. Number in the histogram depicts the MFI.

Figure S7

[A]

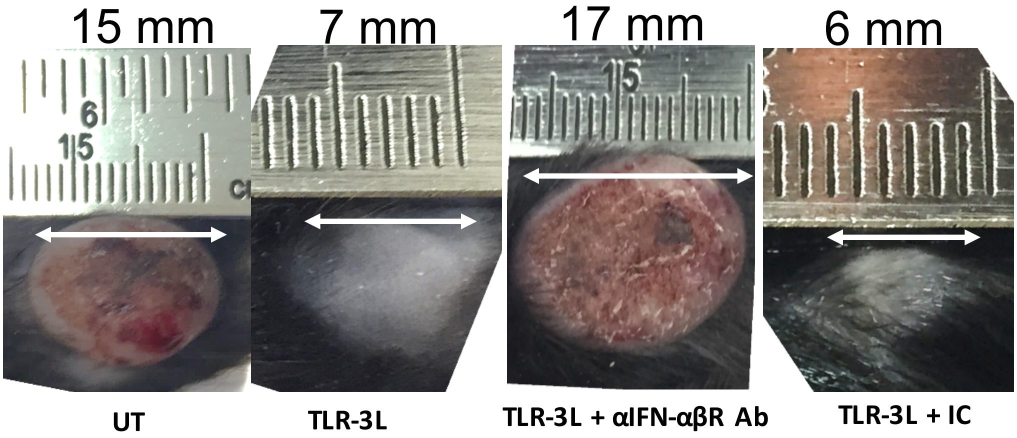

Figure S7. The decrease in the tumor size in the animals administered TLR-3L was through the involvement of IFN-αβR. Tumor bearing mice were injected s.c. with 3 doses of TLR-3L with a gap of 3d. The specificity of the involvement of IFN-αβR was established by injecting blocking Abs against IFN-αβR in the tumor bearing mice. Data shown are representative of 2 independent experiments. The images are representative of MC38 tumors. IC: isotype control.

Figure S8

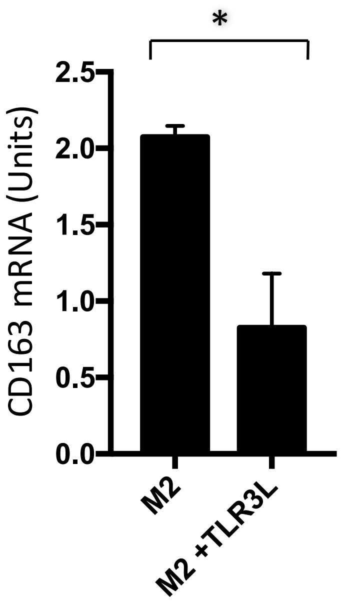

Figure S8. TLR-3 triggering reduced the expression of CD163 on human M2 macrophages. Primary human macrophages were cultured in M2 differentiating conditions followed by treatment with TLR-3L for 24h. The expression of CD163 was monitored by RT-qPCR and depicted as bar diagram. *p<0.05

Figure S9

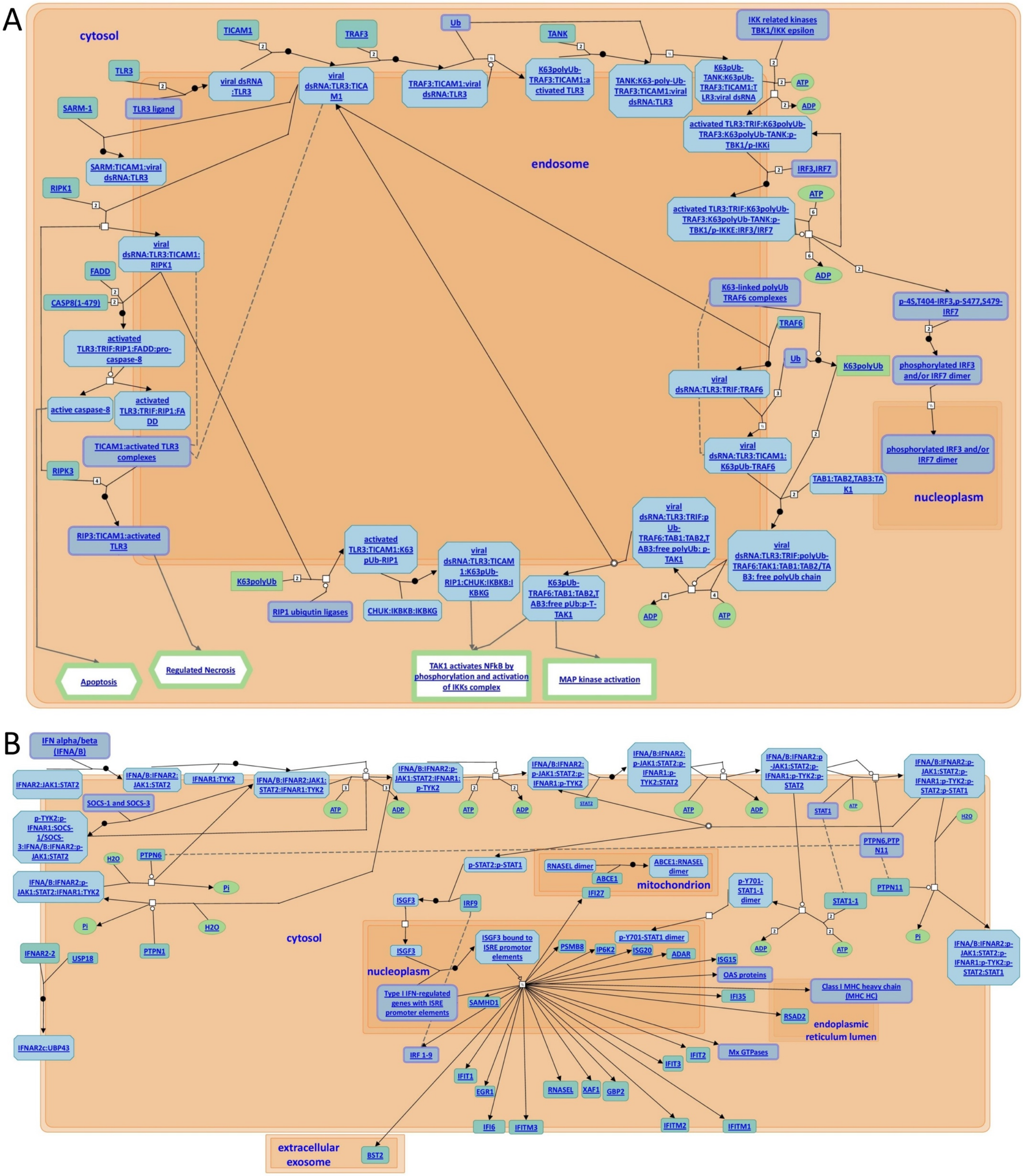
A
cytosol
endosome
nucleoplasm
TICAM1
TRAF3
Ub
TANK
IKK related kinases TBK1/IKK epsilon
TLR3
viral dsRNA :TLR3
viral dsRNA:TLR3:TICAM1
TRAF3:TICAM1:viral dsRNA:TLR3
K63polyUb-TRAF3:TICAM1:activated TLR3
TANK:K63-poly-Ub-TRAF3:TICAM1:viral dsRNA:TLR3
K63pUb-TANK:K63pUb-TRAF3:TICAM1:TLR3:viral dsRNA
ATP
ADP
SARM-1
TLR3 ligand
SARM:TICAM1:viral dsRNA:TLR3
activated TLR3:TRIF:K63polyUb-TRAF3:K63polyUb-TANK:p-TBK1/p-IKKi
IRF3,IRF7
activated TLR3:TRIF:K63polyUb-TRAF3:K63polyUb-TANK:p-TBK1/p-IKKE:IRF3/IRF7
RIPK1
viral dsRNA:TLR3:TICAM1:RIPK1
FADD
CASP8(1-479)
activated TLR3:TRIF:RIP1:FADD:pro-caspase-8
K63-linked polyUb TRAF6 complexes
TRAF6
p-4S,T404-IRF3,p-S477,S479-IRF7
Ub
K63polyUb
viral dsRNA:TLR3:TRIF:TRAF6
phosphorylated IRF3 and/or IRF7 dimer
active caspase-8
activated TLR3:TRIF:RIP1:FADD
TICAM1:activated TLR3 complexes
viral dsRNA:TLR3:TICAM1:K63pUb-TRAF6
phosphorylated IRF3 and/or IRF7 dimer
RIPK3
TAB1:TAB2,TAB3:TAK1
RIP3:TICAM1:activated TLR3
viral dsRNA:TLR3:TRIF:p Ub-TRAF6:TAB1:TAB2,TAB3:free polyUb: p-TAK1
activated TLR3:TICAM1:K63 pUb-RIP1
viral dsRNA:TLR3:TICAM1:K63pUb-RIP1:CHUK:IKBKB:IKBKG
viral dsRNA:TLR3:TRIF:polyUb-TRAF6:TAK1:TAB1:TAB2/TAB3: free polyUb chain
K63polyUb
RIP1 ubiqutin ligases
CHUK:IKBKB:IKBKG
K63pUb-TRAF6:TAB1:TAB2,TAB3:free pUb:p-T-TAK1
ADP
ATP
Apoptosis
Regulated Necrosis
TAK1 activates NFkB by phosphorylation and activation of IKKs complex
MAP kinase activation
B
IFN alpha/beta (IFNA/B)
IFNAR2:JAK1:STAT2
IFNA/B:IFNAR2:JAK1:STAT2
IFNAR1:TYK2
IFNA/B:IFNAR2:JAK1:STAT2:IFNAR1:TYK2
IFNA/B:IFNAR2:p-JAK1:STAT2:IFNAR1:p-TYK2
IFNA/B:IFNAR2:p-JAK1:STAT2:p-IFNAR1:p-TYK2
IFNA/B:IFNAR2:p-JAK1:STAT2:p-IFNAR1:p-TYK2:STAT2
IFNA/B:IFNAR2:p-JAK1:STAT2:p-IFNAR1:p-TYK2:p-STAT2
IFNA/B:IFNAR2:p-JAK1:STAT2:p-IFNAR1:p-TYK2:p-STAT2:p-STAT1
SOCS-1 and SOCS-3
p-TYK2:p-IFNAR1:SOCS-1/SOCS-3:IFNA/B:IFNAR2:p-JAK1:STAT2
ATP
ADP
STAT1
H2O
PTPN6
PTPN6,PTPN11
IFNA/B:IFNAR2:p-JAK1:STAT2:IFNAR1:TYK2
Pi
p-STAT2:p-STAT1
RNASEL dimer
ABCE1:RNASEL dimer
ABCE1
mitochondrion
IFI27
p-Y701-STAT1-1 dimer
PTPN11
STAT1-1
ISGF3
IRF9
cytosol
PTPN1
ISGF3 bound to ISRE promotor elements
PSMB8
IP6K2
ISG20
ADAR
p-Y701-STAT1 dimer
ISG15
OAS proteins
IFNAR2-2
USP18
nucleoplasm
Type I IFN-regulated genes with ISRE promoter elements
SAMHD1
IFI35
Class I MHC heavy chain (MHC HC)
RSAD2
endoplasmic reticulum lumen
IFNAR2c:UBP43
IRF 1-9
IFIT3
IFIT2
Mx GTPases
IFIT1
EGR1
RNASEL
XAF1
GBP2
IFI6
IFITM3
IFITM2
IFITM1
IFNA/B:IFNAR2:p-JAK1:STAT2:p-IFNAR1:p-TYK2:p-STAT2:STAT1
extracellular exosome
BST2

Figure S9 TLR-3 signaling cascade leads to activation of type I interferon transcription factors, which subsequently induces effective innate immune response in humans (A) Analyzing the reactome pathway knowledgebase to identify the interacting partners involved in the downstream signaling events of TLR-3 ligand stimulation shows the up regulation of key transcriptional regulators of type I interferon (IFN-αβ), Interferon regulatory factor 7 (IRF7) and Interferon regulatory factor 3 (IRF3). Phosphorylation, and the subsequent translocation of IRF7 and/or IRF3 into nucleus is essential in synthesis of IFN-αβ by immune cells. (B) Binding of type I interferon on its receptor induces robust innate immune response via JAK-STAT signaling cascade, along with phosphorylation of key mediators for transcription of IFN-stimulated genes (ISG). Major components under the regulation of ISG also involves M1 signature markers, inflammatory response genes, and MHC class I, thus indicating initiation of potent cell mediated immune response. Intracellular proteins and transcription factors involved in the signal transduction events during TLR3 and IFN stimulus represents that of *Homo sapiens*.

Figure S10

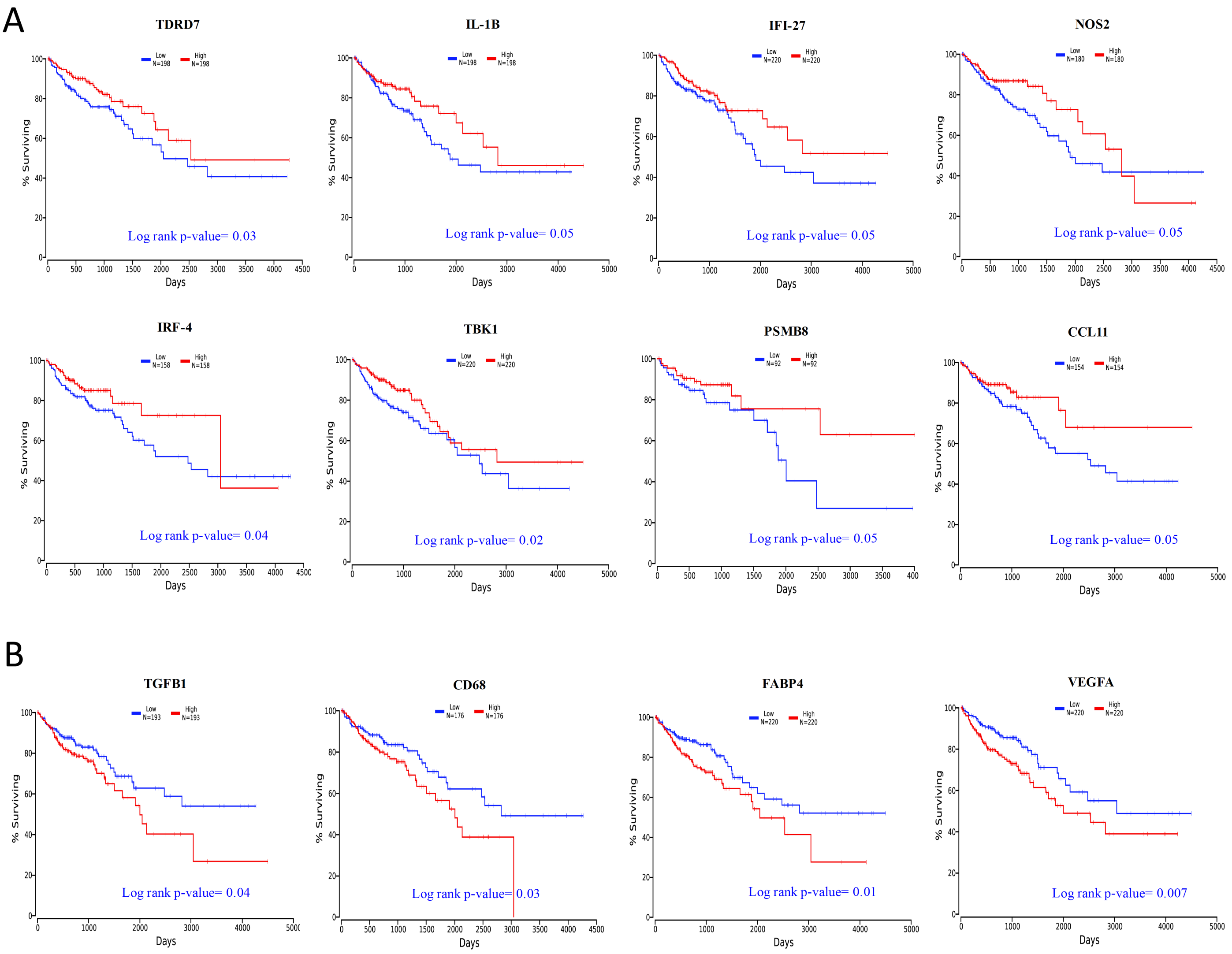

Figure S10 Clinical significance of M1, M2 and TAMs markers on the survival correlations in Colon Adenocarcinoma patients indicates therapeutic potential in reverting M2 macrophages to M1 TGCA recourse portal stores comprehensive information on various cancer types in human patients. OncoLnc tool enabled to explore the survival correlations of the following markers in COAD patient data in TGCA portal. (A) M1 signature markers and cytokines: TDRD7, IL-1β, CCL11; Tumoricidal and inflammatory response genes under regulation of ISG: NOS2, TBK1, IFI-27, PSMB8. (B) M2 and TAM associated genes: TGFβ1, CD68, FABP4, VEGFA. Kaplan-Meier plots depict the survival correlation in COAD patients expressing the above genes, and the level of significance is represented by the log rank p-value (< 0.05). Red and blue lines indicate the cohort of COAD patients with high and low expression of the subject genes respectively.
